# Retraction: LOXL2 upregulation in gliomas drives tumorigenicity by activating autophagy to promote TMZ resistance and trigger EMT

**DOI:** 10.3389/fonc.2024.1475049

**Published:** 2024-08-27

**Authors:** Frontiers Editorial Office

The journal retracts the 29th October 2020 article cited above.

Following publication, concerns were raised regarding the integrity of the images in the published figures. The authors requested retraction due to the image integrity concerns, which was then investigated and conducted in accordance with Frontiers’ policies.

This retraction was approved by the Chief Executive Editor of Frontiers. The authors agreed with the retraction.

